# Pelleting and particle size reduction of corn increase net energy and digestibility of fiber, protein, and fat in corn-soybean meal diets fed to group-housed pigs

**DOI:** 10.1186/s40104-024-01004-9

**Published:** 2024-04-05

**Authors:** Su A Lee, Diego A. Rodriguez, Chad B. Paulk, Hans H. Stein

**Affiliations:** 1 Department of Animal Sciences, University of Illinois, Urbana, IL 61801 USA; 2 Department of Grain Sciences and Industry, Kansas State University, Manhattan, KS 66506 USA

**Keywords:** Corn, Digestibility, Feed technology, Net energy, Particle size, Pelleting

## Abstract

**Background:**

Reduction of the particle size of corn increases energy digestibility and concentrations of digestible and metabolizable energy. Pelleting may also reduce particle size of grain, but it is not known if there are interactions between particle size reduction and pelleting. The objective of this experiment was to test the hypothesis that particle size reduction and pelleting, separately or in combination, increase N balance, apparent total tract digestibility (ATTD) of fiber and fat, and net energy (NE) in corn-soybean meal diets fed to group-housed pigs.

**Methods:**

Six corn-soybean meal-based diets were used in a 3 × 2 factorial design with 3 particle sizes of corn (i.e., 700, 500, or 300 μm) and 2 diet forms (i.e., meal or pelleted). Pigs were allowed ad libitum access to feed and water. Twenty-four castrated male pigs (initial weight: 29.52 kg; standard diviation: 1.40) were allotted to the 6 diets using a 6 × 6 Latin square design with 6 calorimeter chambers (i.e., 4 pigs/chamber) and 6 periods. Oxygen consumption and CO_2_ and CH_4_ productions were measured during fed and fasting states and fecal and urine samples were collected.

**Results:**

Regardless of particle size of corn, the ATTD of gross energy (GE), N, and acid-hydrolyzed ether extract (AEE), and the concentration of NE were greater (*P* < 0.05) in pelleted diets than in meal diets. Regardless of diet form, the ATTD of GE, N, and AEE, and the concentration of NE were increased (linear; *P* < 0.05) by reducing the particle size of corn, but the increase was greater in meal diets than in pelleted diets (interaction; *P* < 0.05).

**Conclusions:**

Both pelleting and reduction of corn particle size increased nutrient digestibility and NE, but increases were greater in meal diets than in pelleted diets.

## Background

Apparent ileal digestibility of starch and gross energy (GE) is increased by reducing particle size of cereal grains because the increase in surface area of grain particles allows for greater interactions with digestive enzymes [1–3]. Improvement in the apparent total tract digestibility (ATTD) of GE upon particle size reduction has also been demonstrated in corn and a number of other ingredients fed to weanling or growing-finishing pigs [4, 5].

Pelleting also increases the ATTD of GE primarily because starch in cereal grains is gelatinized when heat, moisture, and pressure are applied to ingredients during pelleting [5]. Applying heat, moisture, and pressure during the pelleting process also results in the inactivation of anti-nutritional factors and this may increase nutrient digestibility as well [6–8]. However, pelleting may also reduce particle size of grain [9], and it is not known if improvements in nutrient digestibility obtained by reducing the particle size of grain and improvements obtained by pelleting are additive or if there are interactions between particle size reduction and pelleting.

Prediction equations for net energy (NE) in diets fed to pigs based on chemical composition are widely used [10, 11]. However, the equations were developed without considering feed processing including pelleting or particle size. These equations may, therefore, not be applicable to pelleted diets containing ingredients with different particle size, but data to verify this possibility have not been published. Therefore, an experiment was conducted to test the hypothesis that particle size reduction and pelleting, separately or in combination, increase N balance, ATTD of fiber and fat, and NE in corn-soybean meal diets fed to group-housed pigs.

## Methods

The Institutional Animal Care and Use Committee at the University of Illinois reviewed and approved the protocol for the experiment before animal use was initiated. Pigs were the offspring of Line 800 boars and Camborough females (Pig Improvement Company, Hendersonville, TN, USA). The same batches of corn and soybean meal were used to prepare all diets used in the experiment.

### Diet and diet preparation

Dietary treatments were arranged as a 3 × 2 factorial design with 3 particle sizes of corn (i.e., 700, 500, or 300 μm) and 2 diet forms (i.e., meal or pelleted; Tables 1 and 2). Diet manufacturing occurred at the Kansas State University O.H. Kruse Feed Technology and Innovation Center (Manhattan, KS, USA). Corn was ground using a 3-high roller mill (Model 924; RMS, Harrisburg, SD, USA) to an approximate particle size of 700, 500, or 300 μm. Prior to mixing, particle size was determined using a 13-sieve stack with US sieve numbers 6, 8, 12, 16, 20, 30, 40, 50, 70, 100, 140, 200, 270, and pan. A Ro-Tap shaker (W.S. Tyler, Mentor, OH, USA) was used to sift the 100 g samples for 15 min using sieve agitators including bristle sieve cleaners and rubber balls on select sieves. Particle size analysis was conducted with the addition of 0.5 g flow agent (Amorphous silica powder; Gilson Company Inc., Lewis Center, OH, USA) per 100 g sample. Geometric mean particle size by mass (*d*_gw_) and the geometric standard deviation of particle diameter by mass (*S*_gw_) were calculated using the quantity of sample remaining on each screen following the shaking procedure [12, 13]. The analyzed *d*_gw_ were 685, 526, and 320, and *S*_gw_ were 2.61, 2.37, and 2.20 for the 700, 500, or 300 μm treatments, respectively. Feed was then mixed in a 1-ton double ribbon mixer (Hayes and Stolz Industrial Manufacturing Co. LLC, Burleson, TX, USA). Diets were steam conditioned using a 25 cm × 140 cm twin shaft pre-conditioner (Model 150, Wenger, Sabetha, KS, USA) for 30 s at 83 °C, and subsequently pelleted using a 30-horsepower pellet mill (1012-2 HD Master Model, California Pellet Mill, Crawfordsville, IN, USA) equipped with a 31.8 mm × 4.8 mm (L:D 6.6) pellet die. Treatments were pelleted at 15.2 kg/min and had a hot pellet temperature of 88.5 °C. Particle size of meal diets was also measured as for ground corn.

**Table 1:** Ingredient composition of experimental diets (as-fed basis)

Item, %	Diet form	Meal			Pellet		
	**Corn particle size, μm**	**700**	**500**	**300**	**700**	**500**	**300**
Ground corn, 700 μm		78.100	-	-	78.100	-	-
Ground corn, 500 μm		-	78.100	-	-	78.100	-
Ground corn, 300 μm		-	-	78.100	-	-	78.100
Soybean meal		17.150	17.150	17.150	17.150	17.150	17.150
Soybean oil		1.500	1.500	1.500	1.500	1.500	1.500
Dicalcium phosphate		0.891	0.891	0.891	0.891	0.891	0.891
Ground limestone		0.700	0.700	0.700	0.700	0.700	0.700
L-Lys·HCl (78.8% Lys)		0.272	0.272	0.272	0.272	0.272	0.272
DL-Met (99% Met)		0.024	0.024	0.024	0.024	0.024	0.024
L-Thr (99% Thr)		0.063	0.063	0.063	0.063	0.063	0.063
Sodium chloride		0.400	0.400	0.400	0.400	0.400	0.400
Titanium dioxide		0.400	0.400	0.400	0.400	0.400	0.400
Vitamin-micromineral premix^a^		0.500	0.500	0.500	0.500	0.500	0.500

^a^The vitamin-micromineral premix provided the following quantities of vitamins and micro minerals per kg of complete diet: vitamin A as retinyl acetate, 10,622 IU; vitamin D_3_ as cholecalciferol, 1,660 IU; vitamin E as DL-alpha-tocopheryl acetate, 66 IU; vitamin K as menadione nicotinamide bisulfate, 1.40 mg; thiamin as thiamine mononitrate, 1.08 mg; riboflavin, 6.49 mg; pyridoxine as pyridoxine hydrochloride, 0.98 mg; vitamin B_12_, 0.03 mg; D-pantothenic acid as D-calcium pantothenate, 23.2 mg; niacin, 43.4 mg; folic acid, 1.56 mg; biotin, 0.44 mg; Cu, 20 mg as copper chloride; Fe, 123 mg as iron sulfate; I, 1.24 mg as ethylenediamine dihydriodide; Mn, 59.4 mg as manganese hydroxychloride; Se, 0.27 mg as sodium selenite and selenium yeast; and Zn, 124.7 mg as zinc hydroxychloride

**Table 2:** Analyzed nutrient composition of experimental diets (as-fed basis)

Item, %	Diet form	Meal			Pellet		
	**Corn particle size, μm**	**700**	**500**	**300**	**700**	**500**	**300**
Dry matter		89.51	89.51	89.44	90.12	90.55	89.92
Crude protein		13.46	13.06	12.90	12.30	12.77	13.20
Acid-hydrolyzed ether extract		4.48	4.43	4.57	5.07	5.16	5.10
Ash		4.20	4.16	4.11	4.00	4.08	4.07
Gross energy, kcal/kg		3,920	3,876	3,917	3,951	3,976	3,958
Total dietary fiber		10.9	9.1	10.1	10.5	9.7	10.5
Soluble dietary fiber		0.3	0.6	0.4	0.7	0.5	1.0
Insoluble dietary fiber		10.6	8.5	9.7	9.8	9.2	9.5
Corn particle size (*d*_*gw*_^a^), μm		685	526	320	685	526	320
Standard deviation (*S*_*gw*_^a^)		2.61	2.37	2.20	2.61	2.37	2.20
Diet particle size (*d*_*gw*_), μm		704	557	394	-	-	-
Standard deviation (*S*_*gw*_)		2.36	2.47	2.23	-	-	-

^a^*d*_*gw*_ Geometric mean particle diameter by mass, *S*_*gw*_ Geometric standard deviation of particle diameter by mass

### Animals, housing, and sample collection

Twenty-four growing pigs [initial body weight (BW): 29.52 kg; standard deviation: 1.40] were housed in a calorimeter unit with 6 chambers and 4 pigs per chamber with 2 barrows and 2 gilts. The 6 diets were used in the 6 chambers according to a 6 × 6 Latin square design with 6 periods. Therefore, there were 6 replicate chambers per diet. Each chamber was equipped with a stainless steel wet-dry feeder, and an auxiliary nipple waterer to ensure free access to water. Each chamber had a fully slatted tribar floor, and fecal screens and urine pans were installed below the slatted floor, which allowed for total, but separate, collection of fecal and urine materials. The temperature and relative humidity inside the chambers were controlled by a temperature and humidity control unit at 23 °C and 55%, respectively (Model 9241-2220-B1D0000; Parameter, Black Mountain, NC, USA), and air velocity was controlled using an airflow meter (AccuValve; Accutrol, LLC, Danbury, CT, USA). The final BW of pigs was 111.33 kg (standard deviation: 3.50).

Throughout the experiment, except during fasting periods, pigs were allowed ad libitum access to feed. Diets were fed for 13 d, where the initial 7 d were considered the adaptation period to the diet. At 0700 h on d 8, gas analyzers (*Classic Line*, Sable System Int., North Las Vegas, NV, USA) started measuring O_2_ consumption and CO_2_ and CH_4_ productions and gas measurements ceased at 0700 h on d 14. Total collection of fecal and urine samples was also initiated on d 8 in the morning and ceased on d 14 in the morning and this time was considered the fed period and fecal and urine samples were, therefore, collected on a time basis. Starting at 0700 h on d 14, pigs were deprived of feed for 36 h. This time was considered the fasting period. The initial 24 h of fasting were considered the time the animals digested and metabolized the remaining feed in the intestinal tract to produce energy and gas consumption and production were not measured during this time. However, the last 12 h of the fasting period were considered the actual time where animals mobilized endogenous nutrients to produce energy and gas consumption and production were measured during this time and urine samples were collected quantitatively to calculate fasting heat production (FHP). Therefore, each period lasted 14.5 d.

To avoid N loss from the urine, 100 mL of 6 mol/L HCl were added to each urine pan every day during collection periods. Chambers were opened every day to check feeders, and for collection of fecal and urine materials during the collection period. Data from the gas analyzers obtained during the time chambers were open and until they reached the condition set by the temperature and humidity control unit were disregarded in the final calculation of heat production. Fecal samples and 5% of the collected urine were stored at −20 °C immediately after collection.

At the end of each period, fecal samples were thawed and mixed within chamber and diet, and then dried in a 50 °C forced-air drying oven (Heratherm OMH750; Thermo Fisher Scientific Inc., Waltham, MA, USA). Fecal samples were ground through a 1-mm screen using a hammer mill (model: MM4; Schutte Buffalo, NY, USA). Urine samples were thawed and mixed within chamber and diet for fed and fasting periods, respectively. To prepare urine for GE analysis, urine subsamples were mixed and 10 mL of urine was dripped onto cotton balls that were placed in a plastic bag and lyophilized [14]. A second subsample of urine from each chamber and period was stored at −20 °C without being lyophilized. These samples were analyzed for N.

### Chemical analysis

All nutrients in samples were analyzed in duplicate. Diets, dried and ground fecal samples, and lyophilized urine samples were analyzed for GE using an isoperibol bomb calorimetry (Model 6400; Parr Instruments, Moline, IL, USA). Diet and fecal samples were analyzed for dry matter (DM; method 930.15 [15]), and ash in diet samples was analyzed as well (method 942.05 [15]). Nitrogen in diets and fecal samples was determined using a LECO FP628 Nitrogen Analyzer (LECO Corp., Saint Joseph, MI, USA; method 990.03 [15]) and crude protein was calculated as analyzed N × 6.25. However, N in urine samples from both fed and fasting periods was determined using a Kjeltec 8400 apparatus (method 984.13 [15]; FOSS, Eden Prairie, MN, USA). Urine samples that had not been lyophilized were used for this analysis. In previous work we have compared results of the 2 procedures and not observed significant differences between the combustion and Kjeldahl methods.

Acid-hydrolyzed ether extract (AEE) in diet and fecal samples was analyzed by acid hydrolysis using 3 mol/L HCl (AnkomHCl; Ankom Technology, Macedon, NY, USA) followed by crude fat extraction using petroleum ether (method 2003.06 [15]) on an Ankom fat analyzer (AnkomXT^15^; Ankom Technology, Macedon, NY, USA). Diet and fecal samples were analyzed for insoluble dietary fiber and soluble dietary fiber using the Ankom Dietary Fiber Analyzer (Ankom Technology, Macedon, NY, USA; method 991.43 [15]) and total dietary fiber (TDF) was calculated as the sum of insoluble and soluble dietary fiber.

### Calculations

The ATTD of GE, DM, TDF, and AEE was calculated for each diet using the following equation:

$$\mathrm{ATTD}= 100 \times [(\mathrm{intake }-\mathrm{ excretion}) /\mathrm{ intake}]$$

where intake and excretion were expressed as kcal/d or g/d and excretion refer to fecal excretion. Concentrations of digestible energy (DE) and metabolizable energy (ME) in each diet were calculated as well [11]. Nitrogen intake, N excretions, and the ATTD and retention of N were also calculated.

For NE calculations, concentrations of O_2_, CO_2_, and CH_4_ were averaged separately for the fed period and for the last 12 h of the fasting period. The respiratory quotient was calculated as the ratio between CO_2_ production (L/d) and O_2_ consumption (L/d) according to Richardson [16]. Total heat production (THP) from pigs fed diets during the fed period was calculated using the following equation [17]:

$$\text{THP},\mathrm{kcal}=(3.866\times{\text{O}}_2+1.200\times{\text{CO}}_2-0.518\times{\text{CH}}_4-1.431\times\mathrm{urineN}),$$

where O_2_, CO_2_, and CH_4_ were expressed as L, and urine N was expressed as g. The FHP, which was calculated from pigs during the last 12 h of the fasting period, was calculated as described for THP. Heat increment was calculated by subtracting FHP from THP and NE was then calculated (modified from [11]):

$${\text{NE}},\mathrm{ kcal}/\mathrm{kg }=\mathrm{ ME }- (\mathrm{THP }-\mathrm{ FHP})/\mathrm{intake},$$

where intake refers to average daily feed intake (kg), ME is in kcal/kg, and THP and FHP are in kcal. Energy metabolizability and utilization (%) was calculated by dividing concentration of ME by DE or concentration of NE by ME in diets and multiplying the calculated ratios by 100.

### Statistical analysis

Homogeneity of the variances and normality were confirmed and data were analyzed using the PROC MIXED in SAS (SAS Inst. Inc., Cary, NC, USA). The statistical model included diet as the fixed effect and chamber and period as the random effects. Contrast statements were used to test effects of diet form, linear effects of particle size, and the interaction between diet form and particle size. Mean values were calculated using the LSMeans statement. A calorimeter chamber was the experimental unit. Results were considered significant at *P* < 0.05 and considered a trend at 0.05 ≤ *P* < 0.10.

## Results

Pigs remained healthy during the experiment and feed refusals were not observed, but orts were collected and dried to calculate the weights of feed wastage. No interactions between diet form and particle size of corn were observed for feed intake and GE intake of pigs, urine GE output, ME intake, THP, FHP, retained energy, respiratory quotient during the fed period, and NE:ME (Table 3). Weight of orts and the ATTD of DM and GE increased with reduced particle size of corn in meal diets, but not in pelleted diets (interaction; *P* < 0.05). Weight of feces and fecal GE output were reduced by reducing particle size of corn in diets, but the effects were greater in meal diets than in pelleted diets (interaction; *P* < 0.05). Likewise, concentrations of DE, ME, and NE were increased by reducing particle size of corn, but more so in meal diets than in pelleted diets (interaction; *P* < 0.05). Regardless of particle size of corn, the ATTD of DM and GE, concentrations of DE, ME, and NE, and ME:DE were greater (*P* < 0.05) in pelleted diets compared with meal diets, but regardless of diet form, the ATTD of DM and GE and concentrations of DE, ME, and NE were linearly increased (*P* < 0.05) by reducing particle size of corn.

**Table 3:** Effects of diet form and particle size of corn on apparent total tract digestibility (ATTD) of dry matter (DM) and gross energy (GE) and concentrations of digestible energy (DE), metabolizable energy (ME), and net energy (NE) in diets and total heat production (THP) and fasting heat production (FHP) from group-housed pigs^a^ (as-is basis)

Item	Diet form	Meal			Pellet			SEM^b^	Contrast *P*-value		
	Corn particle size, μm	700	500	300	700	500	300		Diet form	Particle size^c^	Interaction
Intake											
Feed intake, kg/d		2.72	2.62	2.73	2.66	2.61	2.60	0.18	0.254	0.684	0.197
Orts, kg/d		0.08	0.08	0.09	0.04	0.03	0.04	0.01	< 0.001	0.328	< 0.001
GE intake, Mcal/d		10.66	10.15	10.69	10.53	10.36	10.28	0.72	0.628	0.693	0.340
Fecal excretion											
Dry feces output, kg/d		0.27	0.23	0.23	0.23	0.20	0.22	0.02	0.001	0.010	0.002
GE in feces, kcal/kg		4,539	4,568	4,147	4,087	3,988	3,905	61	< 0.001	< 0.001	< 0.001
Fecal GE output, kcal/d		1,244	1,032	962	924	807	840	60	< 0.001	< 0.001	< 0.001
ATTD of DM, %		89.87	91.25	91.39	91.38	92.23	91.69	0.38	0.004	0.019	0.019
ATTD of GE, %		88.26	89.70	90.89	91.08	92.22	91.79	0.45	< 0.001	< 0.001	< 0.001
Urine excretion											
Urine output, kg/d		6.11	5.90	6.81	4.87	5.22	5.68	0.56	0.001	0.034	0.002
GE in urine, kcal/kg		33.23	33.01	29.19	34.91	35.23	35.05	2.27	0.049	0.318	0.061
Urine GE output, kcal/d		206	197	194	170	183	194	22	0.075	0.583	0.108
Heat increment^d^											
ME intake, kcal/BW^0.6^/d		755	725	778	757	763	749	43	0.814	0.727	0.537
O_2_ consumption, L/d		35.64	34.65	34.81	35.74	35.55	35.38	1.90	0.239	0.275	0.530
CO_2_ production, L/d		35.62	35.55	34.87	36.36	35.90	35.96	2.97	0.091	0.266	0.083
CH_4_ production, L/d		-0.05	-0.04	-0.07	-0.06	-0.08	-0.04	0.05	0.479	0.942	0.839
THP, kcal/d		4,314	4,216	4,218	4,346	4,315	4,299	254	0.166	0.250	0.361
THP, kcal/BW^0.6^/d		358	342	345	350	356	355	23	0.182	0.440	0.759
FHP, kcal/ BW^0.6^/d		212	201	203	207	218	221	16	0.019	0.588	0.196
Retained energy, kcal/d		4,892	4,710	5,313	5,090	5,053	4,946	477	0.782	0.593	0.744
Respiratory quotient, fasted		0.65	0.69	0.67	0.69	0.67	0.71	0.04	0.129	0.293	0.022
Respiratory quotient, fed		1.01	1.03	1.00	1.02	1.03	1.03	0.05	0.469	0.868	0.306
Energy in diets, kcal/kg											
DE		3,459	3,477	3,560	3,599	3,667	3,633	18	< 0.001	< 0.001	< 0.001
ME		3,385	3,402	3,488	3,535	3,596	3,559	18	< 0.001	< 0.001	< 0.001
NE		2,735	2,739	2,838	2,857	2,957	2,926	60	< 0.001	0.015	0.004
Energy utilization, %											
ME:DE		97.85	97.87	97.98	98.23	98.06	97.98	0.15	0.045	0.601	0.098
NE:ME		80.79	80.50	81.35	80.81	82.24	82.19	1.57	0.165	0.200	0.564

^a^Each least squares mean represents 6 observations

^b^*SEM* Standard error of the mean

^c^Linear effects of particle size of corn

^d^*BW* Body weight

No interactions between diet form and particle size were observed for N intake, absorbed N, N excretion in urine, and N retention (Table 4). Fecal N excretion was reduced and the ATTD of N was increased by reducing particle size of corn, but the effect was greater in meal diets than in pelleted diets (interaction; *P* < 0.05). Regardless of particle size of corn, fecal N excretion was greater (*P* = 0.001) in meal diets than in pelleted diets, which resulted in greater ATTD of N (*P* = 0.002) in pelleted diets than in meal diets. However, diet form did not affect N retention. Regardless of diet form, the ATTD of N was linearly increased (*P* = 0.031) by reducing particle size of corn. There were no effects of reducing particle size of corn on retention of N in meal or pelleted diets.

**Table 4:** Effects of diet form and particle size of corn on N balance and apparent total tract digestibility (ATTD) of total dietary fiber (TDF) and acid-hydrolyzed ether extract (AEE) in diets fed to group-housed pigs^a^

Item	Diet form	Meal			Pellet			SEM^b^	Contrast *P*-value		
	Corn particle size, μm	700	500	300	700	500	300		Diet form	Particle size^c^	Interaction
N balance											
N intake, g/d		56	52	55	54	53	54	3.73	0.618	0.931	0.313
N in feces, %		3.40	3.51	3.39	3.32	3.28	3.21	0.13	0.093	0.606	0.267
N excretion in feces, g/d		9.29	7.91	7.93	7.49	6.63	6.89	0.55	0.001	0.043	0.005
Absorbed N, g/d		46.38	44.32	47.24	46.09	46.77	47.44	3.45	0.516	0.459	0.977
ATTD of N, %		83.19	84.56	85.49	85.63	87.56	87.25	0.97	0.002	0.031	0.021
N in urine, %		0.19	0.24	0.16	0.24	0.22	0.21	0.05	0.163	0.203	0.029
N excretion in urine, g/d		12.53	15.97	11.55	12.00	12.15	13.21	3.54	0.526	0.944	0.743
N retention, g/d		33.85	28.35	35.68	34.09	34.63	34.22	2.50	0.414	0.697	0.809
N retention, % intake		61.81	56.42	65.50	64.79	65.86	64.47	5.48	0.181	0.620	0.775
N retention, % absorbed		74.48	66.80	76.55	75.92	75.30	73.98	6.72	0.421	0.985	0.879
TDF digestibility											
TDF intake, g/d		296	238	276	280	253	273	18.62	0.771	0.065	0.188
TDF in feces, %		40.07	39.30	42.75	45.17	42.10	45.65	1.72	0.007	0.306	0.014
TDF excretion in feces, g/d		110	91	100	102	86	99	9.20	0.398	0.314	0.503
Absorbed TDF, g/d		186	148	175	177	167	174	14.28	0.706	0.479	0.601
ATTD of TDF, %		62.71	61.85	63.48	63.07	66.30	63.75	2.52	0.395	0.765	0.896
AEE digestibility											
AEE intake, g/d		122	116	125	135	134	133	8.89	< 0.001	0.940	0.006
AEE in feces, %		14.53	16.13	12.09	9.86	10.92	9.20	0.63	< 0.001	0.019	< 0.001
AEE excretion in feces, g/d		40	36	28	22	22	20	2.43	< 0.001	0.002	< 0.001
Absorbed AEE, g/d		82	80	97	113	112	113	7.49	< 0.001	0.053	< 0.001
ATTD of AEE, %		67.12	68.53	77.13	83.33	83.60	84.97	1.45	< 0.001	< 0.001	< 0.001

^a^Each least squares mean represents 6 observations

^b^*SEM* Standard error of the mean

^c^Linear effects of particle size of corn

There was no interaction between diet form and particle size for TDF intake, TDF excretion in feces (g/d), absorbed TDF, or the ATTD of TDF, and these parameters were not affected by diet form or particle size of corn. Excretion of AEE in feces was decreased and absorption and the ATTD of AEE were increased by reducing particle size of corn in meal diets, but not in pelleted diets (interaction; *P* < 0.001). Regardless of particle size of corn, AEE excretion was greater (*P* < 0.001) and AEE intake, absorbed AEE, and the ATTD of AEE were less (*P* < 0.001) in meal diets compared with pelleted diets. Intake of AEE was not affected by particle size of corn regardless of diet form, but AEE excretion in feces was linearly reduced (*P* = 0.002), absorbed AEE tended to be increased (*P* = 0.053), and the ATTD of AEE was increased (*P* < 0.001) by reducing particle size of corn in pelleted and meal diets.

## Discussion

In the U.S., it is recommended that corn be ground to a medium particle size of 600 to 700 μm [5, 9, 18, 19], which was the reason 700 μm corn was used as the coarsest particle size in this experiment. Measured particle size of corn used in each diet and meal diets agreed with expected values. The particle size of the meal diets was slightly different from the particle size of each corn because all diets contained other feed ingredients, primarily soybean meal, with different particle sizes.

The analyzed concentrations of GE, TDF, and AEE in all diets and concentrations of DE and ME in the meal diet containing corn ground to 700 μm were in agreement with calculated values [11], whereas NE in diets, THP, FHP, and NE:ME were greater compared with previous values [10, 11, 20, 21]. Total heat production includes heat productions from heat increment, activity, and for maintaining body temperature [11]. The greater THP obtained in this experiment compared with previous experiments is likely a result of the ad libitum feeding used in this experiment whereas pigs were limit fed in previous experiments. It is also possible that because pigs in this experiment were group-housed rather than housed individually they had increased physical activity, and therefore, greater THP and FHP. However, because growing pigs kept under commercial conditions are group housed and allowed ad libitum intake of feed, it is likely that the THP and FHP measured in this experiment reflect commercial conditions. The increased FHP by pigs resulted in increased NE in diets, and therefore, increased NE:ME. Fasting heat production is affected by previous intake of feed because an increased intake of energy and protein increases the weight of the gastrointestinal tract and liver, which utilize up to 30% of FHP [11, 22–24]. It is, therefore, possible that pigs from this experiment produced more heat during the fasting period because they were allowed ad libitum intake of feed before the fasting period.

The observations that the ATTD of GE, N, and AEE, and concentrations of DE and ME in diets were increased by pelleting agree with previous data [6, 8, 19, 25]. Pelleting is often used in commercial feeding of pigs because it prevents segregation of ingredients, increases bulk density, improves handling and transportation, reduces dust and feed wastage, and improves palatability [26, 27]. There are limited data on effects of pelleting on NE in diets, but the increased concentration of NE in the pelleted diets determined in this experiment is a consequence of the increased ATTD of nutrients and energy that is usually observed in pelleted diets [4, 28]. The increased ATTD of GE and the increased metabolizability of DE (i.e., ME:DE) in the pelleted diets contributed to the increase in NE in the diets because NE:ME was not affected by pelleting. It is possible that fiber is solubilized by pelleting, but ATTD of TDF was not affected by pelleting, indicating that the increased ATTD of GE was not a result of increased fiber digestibility. In contrast, the increased digestibility of AEE and N in the pelleted diets contributed to the increased ATTD of GE. It is also possible that pelleting increased ileal digestibility of starch due to increased gelatinization, but because starch digestibility was not determined we cannot confirm this hypothesis.

The increased ATTD of GE and AEE and increased DE and ME in diets that were observed as particle size of corn was reduced agree with previous data [2, 5, 19, 29, 30]. To our knowledge, effects on NE of reducing particle size of corn fed to pigs have not been reported, but NE of oats increased as particle size was reduced [31]. The surface area is increased by reducing the particle size of feed ingredients, which allows feed particles to better interact with digestive enzymes in the intestinal tract of pigs compared with coarse particles [29]. Therefore, digestibility of nutrients and energy is often increased by reducing particle size, which was also observed in this experiment. It therefore appears that the increase in DE, ME, and NE in diets that was observed as particle size was reduced was a result of increased digestibility of nutrients rather than improved post-absorptive metabolism.

Net energy in diets or ingredients are often estimated using prediction equations based on chemical composition [10, 11]. However, these equations do not consider particle size or whether diets were fed in a meal form or as pellets. Consequently, these equations may not be suitable for pelleted diets containing ingredients with different particle sizes. The results from this experiment confirmed this limitation.

Results of experiments aimed at determining effects of reducing particle size on digestibility of fiber have been inconsistent. The ATTD of neutral detergent fiber and acid detergent fiber was increased if whole diets were ground to fine particles [32], but the ATTD of neutral detergent fiber in corn was reduced by reducing particle size [33]. However, the ATTD of hemicellulose was increased by particle size reduction [33, 34], whereas the ATTD of neutral detergent fiber and acid detergent fiber was the greatest if particle size of corn was approximately 600 μm [29, 30]. Reducing particle size may solubilize fiber and increase fermentation because the increased surface area allows microbes better access to the substrates [35]. However, these effects were not big enough to influence the ATTD of TDF in this experiment.

One of the reasons NE was increased more by particle size reduction of meal diets than of pelleted diets was the increased ATTD of AEE in meal diets, but not in pelleted diets. However, all pelleted diets had greater ATTD of AEE than the meal diets, which likely contributed to the greater NE in pelleted diets. It therefore appears that both pelleting or reduced particle size contribute to an increased ATTD of AEE, but effects are not additive because effects are less in pelleted diets than in meal diets.

In the present experiment, group-housed pigs that were allowed ad libitum access to feed were used as is the case for commercially fed pigs. In contrast, in most previous NE work with pigs, individually housed pigs fed a restricted amount of feed were used [10, 20, 21]. It has, however, been demonstrated that ATTD of GE and DE in restricted fed pigs is greater than in pigs allowed ad libitum access to feed [36], and when the objective is to replicate commercial conditions it is, therefore, more accurate to use pigs allowed ad libitum intake of feed when determining NE values. Indeed, in recent work with broiler chickens, group housed birds with free access to feed were used [37, 38] further indicating that this approach may be more practical in determination of NE values that are aimed at being applied to commercially fed animals.

## Conclusions

Pelleting increased the ATTD of DM, GE, N, and AEE in diets fed to pigs, which resulted in increased NE in pelleted diets compared with meal diets. Reducing particle size of corn increased the ATTD of DM, GE, N, and AEE, and also increased NE. Effects of particle size of corn on improving digestibility of nutrients and concentration of NE were greater if particle size of corn was reduced in meal diets than if particle size of corn was reduced in pelleted diets. However, the absolute values were greater in pelleted diets, which may be a result of particle size also being reduced by pelleting.

## Data Availability

The datasets used and/or analyzed during the current study are available from the corresponding author upon reasonable request.

## Abbreviations

AEE: Acid-hydrolyzed ether extract
ATTD: Apparent total tract digestibility
BW: Body weight
DE: Digestible energy
*d*_*gw*_: Geometric mean particle diameter by mass
DM: Dry matter
FHP: Fasting heat production
GE: Gross energy
ME: Metabolizable energy
NE: Net energy
*S*_*gw*_: Geometric standard deviation of particle diameter by mass
TDF: Total dietary fiber
THP: Total heat production

## References

[1] Huang C, Zang J, Song P, Fan P, Chen J, Liu D (2015). Effects of particle size and drying methods of corn on growth performance, digestibility and haematological and immunological characteristics of weaned piglets. Arch Anim Nutr.

[2] Rojas OJ, Stein HH (2015). Effects of reducing the particle size of corn grain on the concentration of digestible and metabolizable energy and on the digestibility of energy and nutrients in corn grain fed to growing pigs. Livest Sci.

[3] Rojas OJ, Liu Y, Stein HH (2016). Effects of particle size of yellow dent corn on physical characteristics of diets and growth performance and carcass characteristics of growing–finishing pigs. J Anim Sci.

[4] Rojas OJ, Stein HH (2017). Processing of ingredients and diets and effects on nutritional value for pigs. J Anim Sci Biotechnol.

[5] Lancheros JP, Espinosa CD, Stein HH (2020). Effects of particle size reduction, pelleting, and extrusion on the nutritional value of ingredients and diets fed to pigs: a review. Anim Feed Sci Technol.

[6] Lahaye L, Ganier P, Thibault JN, Riou Y, Sève B (2008). Impact of wheat grinding and pelleting in a wheat–rapeseed meal diet on amino acid ileal digestibility and endogenous losses in pigs. Anim Feed Sci Technol.

[7] Svihus B, Zimonja O (2011). Chemical alterations with nutritional consequences due to pelleting animal feeds: a review. Anim Prod Sci.

[8] Rojas OJ, Vinyeta E, Stein HH (2016). Effects of pelleting, extrusion, or extrusion and pelleting on energy and nutrient digestibility in diets containing different levels of fiber and fed to growing pigs. J Anim Sci.

[9] Vukmirović Đ, Čolović R, Rakita S, Brlek T, Đuragić O, Solà-Oriol D (2017). Importance of feed structure (particle size) and feed form (mash vs. pellets) in pig nutrition – A review. Anim Feed Sci Technol.

[10] Noblet J, Fortune H, Shi XS, Dubois S (1994). Prediction of net energy value of feeds for growing pigs. J Anim Sci.

[11] NRC. Nutrient requirements of swine. 11th rev. ed. Washington, DC, USA: Natl. Acad. Press; 2012.

[12] ASABE. Method of determining and expressing fineness of feed materials by sieving. St. Joseph, MI, USA: American Society of Agricultural and Biological Engineers; 2008.

[13] de Jong JA, DeRouchey JM, Tokach MD, Dritz SS, Goodband RD, Paulk CB (2016). Effects of wheat source and particle size in meal and pelleted diets on finishing pig growth performance, carcass characteristics, and nutrient digestibility. J Anim Sci.

[14] Kim BG, Petersen GI, Hinson RB, Allee GL, Stein HH (2009). Amino acid digestibility and energy concentration in a novel source of high-protein distillers dried grains and their effects on growth performance of pigs. J Anim Sci.

[15] AOAC Int (2019). Official methods of analysis of AOAC Int.

[16] Richardson HB (1929). The respiratory quotient. Physiol Rev..

[17] Brouwer E. Report of sub-committee on constants and factors. In: Blaxter KL, editor. Proceedings of the 3rd EAAP Symposium on Energy Metabolism. London: Academic press; 1965. p. 441–3.

[18] Nemechek JE, Tokach MD, Dritz SS, Goodband RD, DeRouchey JM, Woodworth JC (2016). Effects of diet form and corn particle size on growth performance and carcass characteristics of finishing pigs. Anim Feed Sci Technol.

[19] Wondra KJ, Hancock JD, Behnke KC, Hines RH, Stark CR (1995). Effects of particle size and pelleting on growth performance, nutrient digestibility, and stomach morphology in finishing pigs. J Anim Sci.

[20] Kim JW, Koo B, Nyachoti CM (2018). Net energy content of canola meal fed to growing pigs and effect of experimental methodology on energy values. J Anim Sci.

[21] Lyu Z, Chen Y, Wang F, Liu L, Zhang S, Lai C (2023). Net energy and its establishment of prediction equations for wheat bran in growing pigs. Anim Biosci.

[22] Baldwin RL (1995). Modeling ruminant digestion and metabolism.

[23] Critser DJ, Miller PS, Lewis AJ (1995). The effects of dietary protein concentration on compensatory growth in barrows and gilts. J Anim Sci.

[24] Koong LJ, Nienaber JA, Pekas JC, Yen JT (1982). Effects of plane of nutrition on organ size and fasting heat production in pigs. J Nutr.

[25] Chassé É, Guay F, Létourneau-Montminy M-P (2022). Effect of pelleting on nutrients and energy digestibility in growing pigs fed corn-soybean meal-based diet or diet containing corn distillers dried grains with solubles (cDDGS), wheat middlings, and bakery meal. Can J Anim Sci.

[26] Chae BJ, Han IK (1998). Processing effects of feeds in swine - review. Asian-Australas J Anim Sci.

[27] de Jong JA, DeRouchey JM, Tokach MD, Dritz SS, Goodband RD, Woodworth JC (2016). Evaluating pellet and meal feeding regimens on finishing pig performance, stomach morphology, and carcass characteristics. J Anim Sci.

[28] Lundblad KK, Issa S, Hancock JD, Behnke KC, McKinney LJ, Alavi S (2011). Effects of steam conditioning at low and high temperature, expander conditioning and extruder processing prior to pelleting on growth performance and nutrient digestibility in nursery pigs and broiler chickens. Anim Feed Sci Technol.

[29] Lyu Z, Wang L, Wu Y, Huang C (2020). Effects of particle size and lipid form of corn on energy and nutrient digestibility in diets for growing pigs. Anim Biosci.

[30] Ma D, Zhu T, Yang F, Zhang S, Huang C (2021). Effects of corn particle size on energy and nutrient digestibility in diets fed to young pigs and adult sows. Anim Biosci.

[31] Koo B, Nyachoti CM (2021). Effect of oat particle size on energy and nutrient utilization in growing pigs. J Anim Sci.

[32] Saqui-Salces M, Luo Z, Urriola PE, Kerr BJ, Shurson GC (2017). Effect of dietary fiber and diet particle size on nutrient digestibility and gastrointestinal secretory function in growing pigs. J Anim Sci.

[33] Acosta JA, Petry AL, Gould SA, Jones CK, Stark CR, Fahrenholz AC (2020). Enhancing digestibility of corn fed to pigs at two stages of growth through management of particle size using a hammermill or a roller mill. Transl Anim Sci.

[34] Zhao JB, Zhang G, Dong WX, Zhang Y, Wang JJ, Liu L (2019). Effects of dietary particle size and fiber source on nutrient digestibility and short chain fatty acid production in cannulated growing pigs. Anim Feed Sci Technol.

[35] Stewart ML, Slavin JL (2009). Particle size and fraction of wheat bran influence short-chain fatty acid production in vitro. Br J Nutr.

[36] Chastanet F, Pahm AA, Pedersen C, Stein HH (2007). Effect of feeding frequency on energy and amino acid digestibility by growing pigs. Anim Feed Sci Technol.

[37] Wu S-B, Swick RA, Noblet J, Rodgers N, Cadogan D, Choct M (2019). Net energy prediction and enenrgy efficiency of feed for broiler chickens. Poultry Sci.

[38] Noblet J, Tay-Zar A-C, Wu S-B, Srichana P, Cozannet P, Geraert P-A, Choct M (2024). Re-evaluation of recent research on metabolic utilization of energy in poultry: Recommendations for a net energy system for broilers. Anim Nutr.

